# R-spondin2 signaling is required for oocyte-driven intercellular communication and follicular growth

**DOI:** 10.1038/s41418-020-0547-7

**Published:** 2020-04-27

**Authors:** Marie-Cécile De Cian, Elodie P. Gregoire, Morgane Le Rolle, Simon Lachambre, Magali Mondin, Sheila Bell, Céline J. Guigon, Anne-Amandine Chassot, Marie-Christine Chaboissier

**Affiliations:** 1 Université Côte d’Azur, CNRS, Inserm, iBV, Nice, France; 2 Université de Corte, Corte, France; 3 Université de Bordeaux, UMS 3420 CNRS-US4 Inserm, Pôle d’imagerie photonique, Bordeaux, France; 4 Cincinnati Children’s Hospital Medical Center, Cincinnati, OH USA; 5 Université de Paris, BFA, UMR 8251, CNRS, ERL U1133, Inserm, Paris, France

**Keywords:** Development, Gene regulation

## Abstract

R-spondin2 (RSPO2) is a member of the R-spondin family, which are secreted activators of the WNT/β-catenin (CTNNB1) signaling pathway. In the mouse postnatal ovary, WNT/CTNNB1 signaling is active in the oocyte and in the neighboring supporting cells, the granulosa cells. Although the role of *Rspo2* has been previously studied using in vitro experiments, the results are conflicting and the in vivo ovarian function of *Rspo2* remains unclear. In the present study, we found that *RSPO2/Rspo2* expression is restricted to the oocyte of developing follicles in both human and mouse ovaries from the beginning of the follicular growth. In mice, genetic deletion of *Rspo2* does not impair oocyte growth, but instead prevents cell cycle progression of neighboring granulosa cells, thus resulting in an arrest of follicular growth. We further show this cell cycle arrest to be independent of growth promoting GDF9 signaling, but rather associated with a downregulation of WNT/CTNNB1 signaling in granulosa cells. To confirm the contribution of WNT/CTNNB1 signaling in granulosa cell proliferation, we induced cell type specific deletion of *Ctnnb1* postnatally. Strikingly, follicles lacking *Ctnnb1* failed to develop beyond the primary stage. These results show that RSPO2 acts in a paracrine manner to sustain granulosa cell proliferation in early developing follicles. Taken together, our data demonstrate that the activation of WNT/CTNNB1 signaling by RSPO2 is essential for oocyte-granulosa cell interactions that drive maturation of the ovarian follicles and eventually female fertility.

## Introduction

In the ovary, cell communication between the oocyte and the neighboring somatic cells, or granulosa cells, is essential for follicular growth that will eventually lead to the release of the oocyte during ovulation. In mice, the primordial follicles assemble perinatally and oocytes become individualized and surrounded by flattened granulosa cells [1, 2]. Once formed, most of the primordial follicles enter a resting phase until they are recruited to support an oocyte during the cyclic process of ovulation [3]. Their activation requires an interaction between the granulosa cells and the oocyte driven by the granulosa cell-derived factor KITL (Kit Ligand) and the KIT receptor expressed by the oocytes [4, 5]. Activated primordial follicles then undergo rapid growth including enlargement of the oocytes and proliferation of the granulosa cells [6, 7]. The formation of mature primary follicles begins with a change in the shape of the granulosa cells from flattened to cuboidal cells. This change is initiated by an increase of granulosa cell proliferation that promotes intercellular contacts between adjacent granulosa cells, followed by an extension of the intercellular adhesion to the oocyte surface [8]. With the increased proliferation, the granulosa cells become cuboidal and more packed on the oocyte surface. Furthermore, the increased packing density of the granulosa cells leads them to adopt a columnar shape with the nucleus becoming adjacent to the basal lamina [9]. Follicular growth continues with the formation of a second inner layer of granulosa cells leading to the formation of secondary follicles. Further divisions of granulosa cells give rise to tertiary, antral, and preovulatory follicles with more than 50,000 granulosa cells [10].

During the early phase of growth, the oocyte secretes three glycoproteins, ZP1-3, that contribute to the formation of an extracellular matrix, the zona pellucida, around the oocyte [10]. At that time, direct contact between the granulosa cells and the oocyte is mediated by transzonal projections. These specialized filopodia are elaborated by the granulosa cells upon the oocyte-secreted growth differentiation factor-9 (GDF9), cross the zona pellucida to reach the oocyte [11]. Accordingly, the lack of GDF9 has a dramatic consequence on follicle development; in its absence a second layer of granulosa cells fails to develop and primary follicles remain [12]. Strikingly, the oocyte continues to grow and reaches the size of an antral oocyte capable of resuming meiosis in *Gdf9*^*−/*^^−^ ovaries [13].

R-spondins (RSPO) are secreted proteins that promote the activation of the canonical WNT/β-catenin (CTNNB1) signaling pathway [14, 15]. To be functional, RSPO bind their receptors LGR4/5/6 and RSPO2/3 and can also bind heparan sulfate proteoglycans. They next recruit the transmembrane E3-ubiquitin ligases ZNRF3 or RNF43 into an inhibition complex [16–20]. The inhibition of ZNRF3/RNF43 then leads to the stabilization of CTNNB1, which, in turn, interacts with the transcription factors LEF or TCF to induce the expression of various target genes including modulators of cell cycle progression [21]. WNT/CTNNB1 signaling notably promotes G1 phase progression via upregulation of *Ccnd1* (CyclinD1) and downregulation of *Cdkn2a* (p21) [22–24]. R-spondins can stimulate cell proliferation by potentiating WNT/CTNNB1 signaling as previously shown in the intestine [25]. During development, *Rspo1* is required for ovarian differentiation and is downregulated in the postnatal ovary [26–28]. In contrast, the expression of R-spondin2 (*Rspo2*) has been detected in mice oocytes at birth and *Rspo2* heterozygous loss-of-function female mice gradually lose their fertility from 4 months of age [29–31]. In vitro, treatments of ovarian cultures with recombinant RSPO1, RSPO2, or WNT3A, three enhancers of WNT signaling or IWR1, a WNT inhibitor, promote granulosa cell expansion [31, 32]. Furthermore, treatments with WNT3A or LiCl also result in the formation of abnormal follicles [32]. In vivo, either forced activation of CTNNB1 or postnatal ectopic expression of RSPO1 induces precancerous lesions in ovaries that do not grow larger than the size of an antral follicle, contain few proliferative cells, and evolve into granulosa cell tumors [28, 33]. These data show divergent roles or even opposite effects of WNT signaling in ovaries. To date, the perinatal lethality associated with conditional loss-of-function mutations of *Ctnnb1* (e.g., *Sf1Cre; Ctnnb1*^*fl/fl*^ mice) has precluded to determine the precise contribution of WNT signaling to folliculogenesis [34]. To address the role of RSPO2 and WNT signaling in physiological conditions, we have performed a detailed analysis of the ovarian phenotype of the *Rspo2* loss-of-function mouse model. Here we show that RSPO2 is a critical factor involved in the communication between the oocyte and the granulosa cells. Indeed, this oocyte-secreted factor induces the activation of WNT/CTNNB1 signaling in the granulosa cells allowing their proliferation and hence follicular growth. Consequently, lack of *Rspo2* in the ovary or *Ctnnb1* in granulosa cells leads to blockage of the follicles at the primary stage.

## Results

### *Rspo2* expression is restricted to the oocyte in postnatal ovaries

**Fig. 1:**
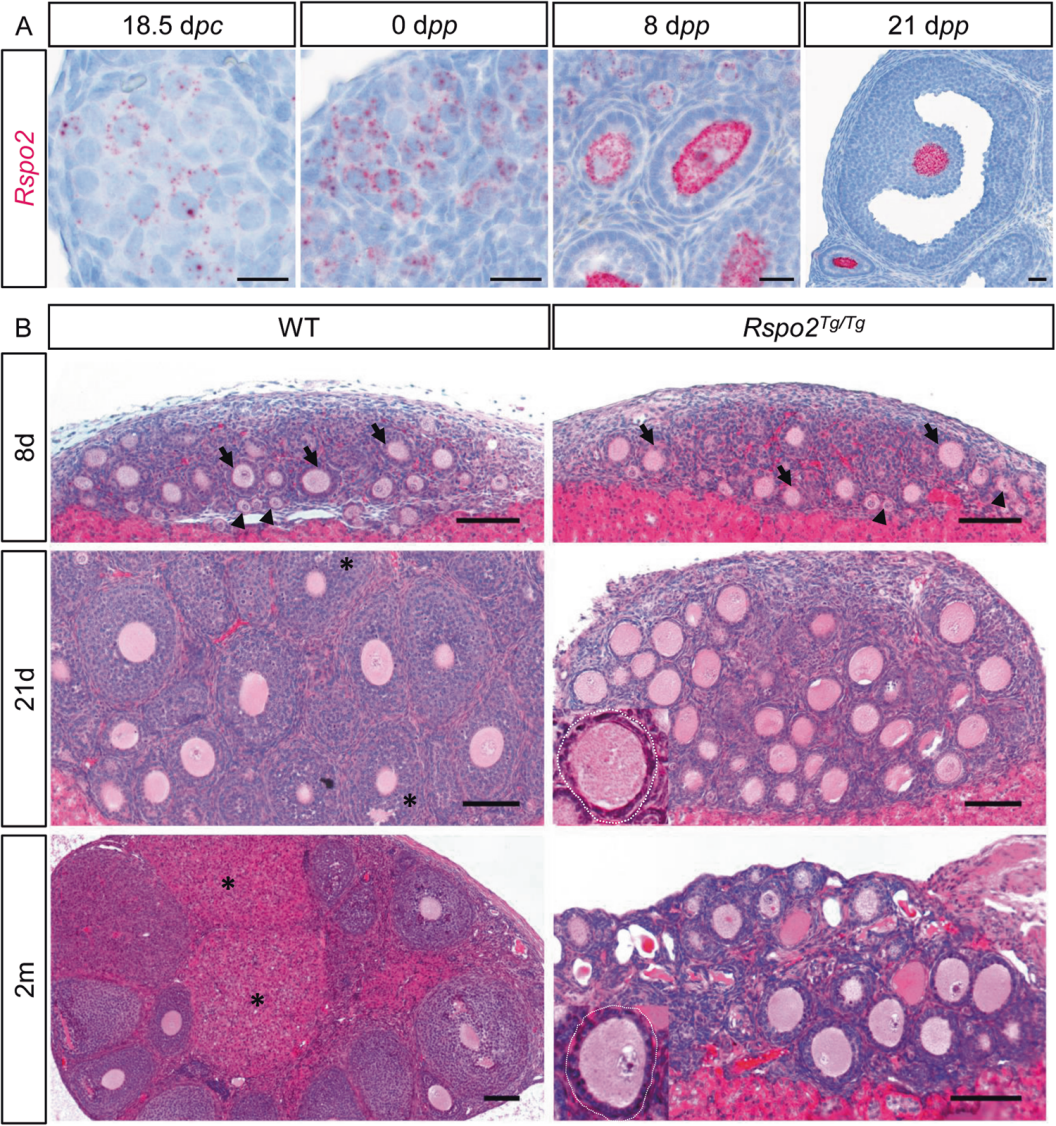
RSPO2 is required for follicular growth. **a** *Rspo2* in situ hybridization analyses at 18.5 d*pc* and 0, 8, and 21 d*pp* show *Rspo2* expression in oocytes after birth in mouse ovaries. Scale bar, 20 µm. **b** Histological analyses (HE staining) of wild-type (WT) and *Rspo2*^*Tg/Tg*^ ovaries at 8 and 21 post-transplantation days (8d, 21d) and 2 months (2m) of 18.5 d*pc* ovaries. WT and *Rspo2*^*Tg/Tg*^ ovaries contain primordial (arrowheads) and primary (arrows) follicles at 8d. At 21d, *Rspo2*^*Tg/Tg*^ ovaries lack secondary, tertiary, and antral (*) follicles, whereas WT ovaries contain these different types of follicles. After 2 months, corpora lutea (*) are observed in WT ovaries. In contrast, follicles remain blocked at the primary–secondary transition in *Rspo2*^*Tg/Tg*^ ovaries. Follicles are outlined with a white dotted line. Scale bars, 100 µm.

To gain insight into the role of *Rspo2* in the ovary, we first characterized the expression pattern of *Rspo2* in mouse ovaries. In situ hybridization experiments revealed that *Rspo2* is weakly expressed in fetal ovaries. At birth (0 d*pp*—day *postpartum*), *Rspo2* expression is restricted to the oocyte of primordial follicles (Fig. 1a). *Rspo2* expression then increases in the oocyte of developing follicles at 8 and 21 d*pp*. Notably, granulosa cells are devoid of *Rspo2* expression irrespective to the time-point considered. In a 12-month-old girl, immunolocalization of RSPO2 demonstrates that RSPO2 is also expressed within the oocyte of primordial, primary, and preantral follicles (Fig. S1a). This suggests that *RSPO2/Rspo2* is expressed in the oocyte as soon as the primordial follicle stage both in humans and mice.

### *Rspo2* loss-of-function ovaries lack secondary follicles

To investigate the function of *Rspo2* in postnatal ovaries, we used a loss-of-function *Rspo2* allele created by a transgene insertional mutation (*Rspo2*^*Tg/Tg*^) [35]. Since the *Rspo2*^*Tg/Tg*^ pups die at birth due to respiratory distress, we transplanted *Rspo2*^*Tg/Tg*^ and wild-type fetal ovaries (18.5 d*pc*—day *post coitum*) under the kidney capsule of athymic female mice. At day 8 post-transplantation (8d), histological analyses revealed that both wild-type and *Rspo2*^*Tg/Tg*^ ovaries contain primordial and primary follicles (Figs. 1b and S1c). This indicates a developmental delay due to transplantation, since non-transplanted wild-type ovaries already harbor secondary follicles at 8 d*pp* (Fig. 1a) [2]. At 12d, wild-type transplanted ovaries contained 45% of secondary follicles (Fig. S1c). At 21d, 36% of the wild-type follicles were tertiary follicles, as evidenced by multiple granulosa cell layers (Fig. S1c). In addition, some follicles had reached the antral stage with the formation of a fluid-filled antrum (Fig. 1b). In contrast, in *Rspo2*^*Tg/Tg*^ ovaries, 62% of the follicles were still at the primary stage with only one layer of nucleus-centered cuboidal granulosa cells (Figs. 1b and S1c). Moreover, some oocytes were surrounded by flattened somatic cells, suggesting that they remained as primordial follicles or were developmentally blocked at the transition from primordial to primary follicle. Others exhibited an asymmetric appearance, with one layer of cells on one side and a disorganized multilayer of granulosa cells on the other side (Fig. S1b). These abnormalities were not observed in wild-type ovaries. Corpora lutea resulting from ovulation were apparent in 2-month-old wild-type transplanted ovaries, whereas *Rspo2*^*Tg/Tg*^ follicles remained blocked as primary follicles (Fig. 1b). In conclusion, our data show that most primary follicles cannot develop beyond early secondary follicles in the absence of *Rspo2*.

### Granulosa cell identity is altered in the absence of RSPO2

**Fig. 2:**
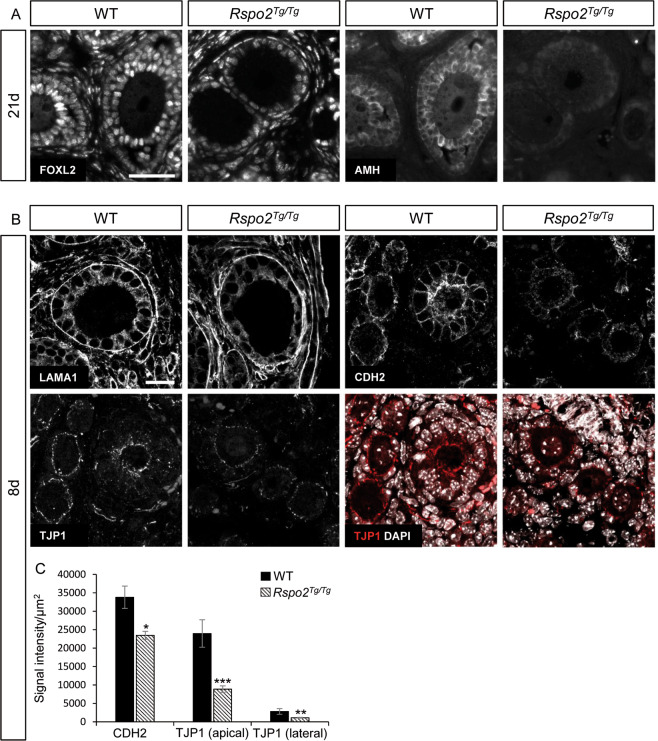
Granulosa cell intercellular junctions are less abundant in *Rspo2*^*Tg/Tg*^ follicles. **a** Immunodetection analyses of FOXL2 and AMH (granulosa cells) at 21d in wild-type (WT) and *Rspo2*^*Tg/Tg*^ transplanted ovaries. In *Rspo2*^*Tg/Tg*^ ovaries, FOXL2 is still expressed in granulosa cells, but AMH expression is impaired compared to wild-type. **b** Immunodetection of LAMA1 (basal lamina), CDH2 (adherens junctions), and TJP1 (tight junctions) at 8d in WT and *Rspo2*^*Tg/Tg*^ transplanted ovaries. Follicles are surrounded by a continuous basal lamina in both genotypes as evidenced by LAMA1 immunostaining. CDH2 and TJP1 signals are decreased, notably at the interface between the oocyte and granulosa cells, indicating a deficit of cohesion in *Rspo2*^*Tg/Tg*^ follicles. Scale bar, 20 µm. **c** This result is corroborated by quantitative analysis of CDH2 and TJP1 signal intensity on confocal sections. Data are presented as mean ± SEM. *n* = 5 WT and *n* = 9 *Rspo2*^*Tg/Tg*^ follicles from one transplanted ovary of each genotype. Student’s *t* test, unpaired two sided (*p < 0.05; **p < 0.01; ***p < 0.001).

Previous data indicate that the transcription factor *Foxl2* is expressed in granulosa and theca cells and is required for the transition from flattened to cuboidal granulosa cells [36, 37]. In *Rspo2*^*Tg/Tg*^ ovaries, FOXL2 immunolocalizations confirmed the identity of the granulosa cells. A subset of these cells also expresses AMH, a marker of granulosa cells in primary to antral follicles [38], but the expression of AMH is sporadic with some cells expressing high levels and others low levels of AMH in *Rspo2*^*Tg/Tg*^ follicles at 12d (Fig. S2a). At 21d, most of the *Rspo2*^*Tg/Tg*^ granulosa cells fail to express AMH but remain FOXL2-positive (Fig. 2a). This indicates that *Rspo2*^*Tg/Tg*^ follicles do not exhibit some hallmarks of primary follicles like AMH expression. FOXL2 immunostainings also revealed some positive stromal cells [39] in *Rspo2*^*Tg/Tg*^ ovaries. These cells were also positive for SF1/NR5A1 indicating that *Rspo2*^*Tg/Tg*^ ovaries contain theca cells (Fig. S2a).

### Intercellular junctions are impaired in *Rspo2*^*Tg/Tg*^ follicles

Given that granulosa cells were disorganized in *Rspo2*^*Tg/Tg*^ primordial/primary follicles (Fig. S1b), we investigated the establishment of the normal granulosa cell polarity. We first analyzed the localization of laminin (LAMA1), a major component of the basal lamina. Immunostainings for LAMA1 did not reveal differences between the wild type and the mutant follicles, indicating a normal deposition of the basal lamina (Fig. 2b). CDH2 (N-Cadherin), present in the adherens junctions of granulosa cells, is robustly expressed from primary follicles onwards [40]. In *Rspo2*^*Tg/Tg*^ follicles, CDH2 was less abundant especially at the interface between oocyte and granulosa cells (Fig. 2b, c). In addition, the tight junction protein TJP1 (ZO1) was found enriched at the interface between oocyte and granulosa cells (apical TJP1), but also present at a lower level between granulosa cells (lateral TJP1) of wild-type ovaries. Immunostainings and quantification analyses highlighted that TJP1 is less expressed in *Rspo2*^*Tg/Tg*^ follicles (Fig. 2b, c). Altogether this shows a defect of the establishment of adherens junctions between the follicular cells and tight junctions at the apical pole of granulosa cells.

### Granulosa cells exhibit cell cycle progression defects in *Rspo2*^*Tg/Tg*^ follicles

**Fig. 3:**
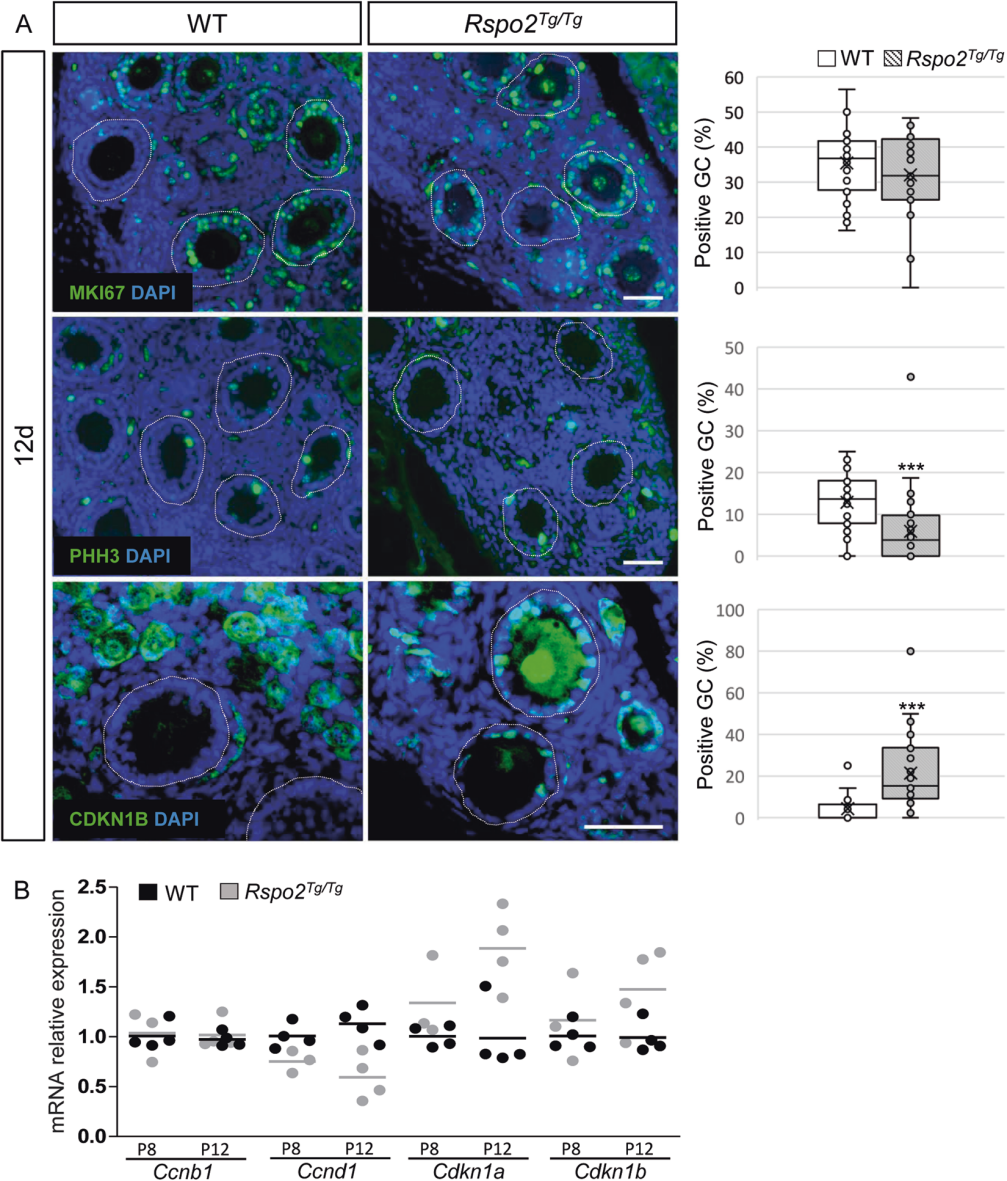
Granulosa cell cycle progression is impaired in the absence of RSPO2. **a** Immunodetection and quantitative analyses of MKI67, PHH3, and CDKN1B (p27) to assess the proliferation status of granulosa cells at 12d in WT and *Rspo2*^*Tg/Tg*^ transplanted ovaries. Left panel: follicular cells are engaged in cell cycle as indicated by MKI67 immunostaining but exhibit an interphase delay illustrated by a decrease of PHH3-positive and an increase of CDKN1B-positive granulosa cells in mutant ovaries. Follicles are outlined with a white dotted line. Right panel: quantification of the % of positive granulosa cells for MKI67, PHH3, and CDKN1B. *n* = 1070, 1107, and 280 WT and *n* = 889, 678, and 583 *Rspo2*^*Tg/Tg*^ granulosa cells (identified with DAPI) from 31, 30, and 10 and 28, 42, and 23 follicles, respectively, from at least two individual transplanted ovaries of each genotype. Data are presented in a box and whisker plot representation to illustrate positive granulosa cell dispersion according to the follicle considered. Student’s *t* test, unpaired two sided (*p < 0.05; **p < 0.01; ***p < 0.001). Scale bar, 50 µm. **b** QRT-PCR analysis of *Ccnb1* (CyclinB1), *CcnD1* (CyclinD1), *Cdkn1a* (p21), and *Cdkn1b* (p27). Both inhibitors of CDK are upregulated, while *Ccnd1* expression is decreased, confirming a reduction of the cell cycle progression in *Rspo2*^*Tg/Tg*^ granulosa cells. Data are presented as individual data points. *n* = 3 (8d) and 4 (12d) individual ovaries per genotype. Mean values are indicated as black (WT) and gray (*Rspo2*^*Tg/Tg*^) bars.

The lack of secondary follicles suggested a defect in proliferation of the granulosa cells in *Rspo2*^*Tg/Tg*^ females. We examined cell cycle progression using MKI67 that is expressed in all active phases of the cell cycle (G1, S, G2, M) at 12d (Fig. 3a). The number of MKI67-positive granulosa cells was similar in wild-type and mutant ovaries, indicating that these cells are proliferating. To evaluate the rate of cell cycle progression, we analyzed the G2/M transition by the quantification of the expression of *Ccnb1* (CyclinB1) and phosphohistone H3 (PHH3) using qRT-PCR and immunolocalization, respectively (Fig. 3a, b). No difference was observed in the level of *Ccnb1* transcripts, but the percentage of PHH3-positive granulosa cells was decreased in *Rspo2*^*Tg/Tg*^ ovaries in comparison with wild types. These observations indicate that fewer granulosa cells attain the G2/M transition phase of the cell cycle in *Rspo2*^*Tg/Tg*^ females. *Ccnd1*, a cyclin involved in G1 phase progression, was downregulated in the *Rspo2*^*Tg/Tg*^ ovary. *Cdkn1a* and *Cdkn1b* (cyclin-dependent kinase inhibitor 1a-b also denoted p21 and p27, respectively), two proteins that mediate cell cycle arrest, were trending to be upregulated at 8d and were confirmed to be upregulated by 12d (Fig. 3b). In addition, immunolocalization of CDKN1B showed an increase of CDKN1B-positive granulosa cells in *Rspo2*^*Tg/Tg*^ females at this stage (Fig. 3a). Altogether, our data indicate that the progression of the granulosa cell cycle is impaired, giving an explanation for the blockage of follicular growth observed in *Rspo2*^*Tg/Tg*^ ovaries. Thus, oocyte-specific RSPO2 appears to be an enhancer of cell cycle progression of granulosa cells during follicular growth.

### Oocyte growth is independent of RSPO2 signaling

Whereas proliferation of the granulosa cells is impaired, the oocytes appear to have a similar size in wild-type and *Rspo2*^*Tg/Tg*^ ovaries at 21d (Figs. 1a and S1b). Before carrying out quantifications, we evaluated the impact of transplantation on follicular growth by comparing the oocyte and the follicular diameters in non-transplanted and transplanted wild-type ovaries at 21 d*pp* and 21d (Fig. S3). This shows that the diameter of the oocyte increases regularly during follicle growth in both conditions (a threefold increase from 20 to 60 µm in tertiary/antral follicles). There is, however, a noticeable difference in the size of antral follicles displaying an average diameter of 250 µm for non-transplanted ovaries and 170 µm for transplanted ovaries. This reflects that the fluid-filled cavity enlargement of antral follicles is less advanced in transplanted ovaries (Fig. 1b). However, our results demonstrate that transplantation does not hamper oocyte and follicle growth.

**Fig. 4:**
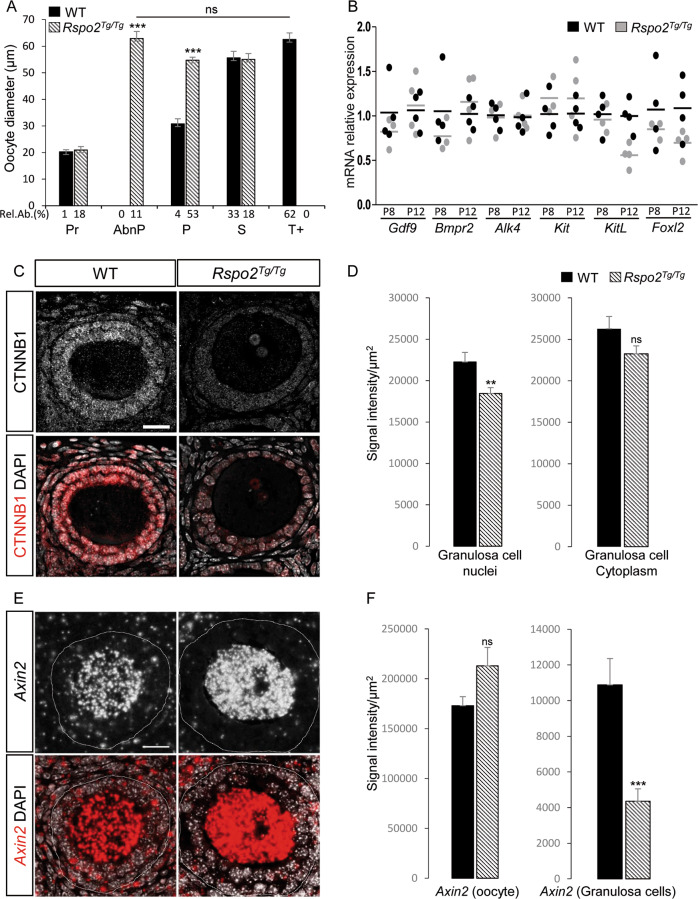
RSPO2 signaling specifically targets granulosa cells. **a** Quantitative analysis of the oocyte diameter size according to follicular stage in WT (*n* = 55; black histogram) and *Rspo2*^*Tg/Tg*^ (*n* = 90; striped histogram) transplanted ovaries at 21d (at least three ovaries of each genotype). Pr primordial, AbnP abnormal primordial *Rspo2*^*Tg/Tg*^ specific follicles, P primary, S secondary, T+ tertiary and antral follicles. Data show that the oocyte growth is not impacted by the absence of *Rspo2* (mean ± SEM). Relative abundance (Rel.Ab.) of each follicular stage is indicated as percentage of total follicles. Student’s *t* test, unpaired two sided (***p < 0.001). **b** QRT-PCR analysis of *Gdf9, Bmpr2*, and *Alk4* (GDF9 receptors); *Kit* receptor (*Kit*); *Kit* ligand (*KitL*); and *Foxl2* expression. Data are presented as individual data points. *n* = 3 (8d) and 4 (12d) individual ovaries per genotype. Mean values are indicated as black (WT) and gray (*Rspo2*^*Tg/Tg*^) bars. **c** Immunodetection and **d** corresponding quantitative analysis of nuclear versus cytoplasmic CTNNB1 in granulosa cells to assess the activation of WNT/CTNNB1 signaling at 12d in wild-type (WT) and *Rspo2*^*Tg/Tg*^ transplanted ovaries (*n* = 20 WT and *n* = 23 *Rspo2*^*Tg/Tg*^ follicles of three ovaries per genotype). In *Rspo2*^*Tg/Tg*^ follicles, CTNNB1 expression in granulosa cell nuclei is significantly decreased. Data are presented as mean ± SEM. Student’s *t* test, unpaired two sided (**p < 0.01). Scale bar, 20 µm. **e** In situ hybridization analysis of *Axin2* expression and **f** signal quantification in WT and *Rspo2*^*Tg/Tg*^ follicles at 12d (*n* = 18 WT and *n* = 14 *Rspo2*^*Tg/Tg*^ follicles of two ovaries per genotype). In *Rspo2*^*Tg/Tg*^ ovaries, *Axin2* expression is unchanged within the oocyte but is decreased in granulosa cells when compared with WT. Data are presented as mean ± SEM. Student’s *t* test, unpaired two sided (***p < 0.001). Scale bar, 20 µm.

Next, we compared the size of oocytes in wild-type and *Rspo2*^*Tg/Tg*^ follicles. At 21d, primordial, primary, secondary, and tertiary/antral follicles constitute 1%, 4%, 33%, and 62% of all wild-type follicles, respectively (Fig. 4a). On average, the oocyte diameter increases from 20 µm in primordial to 30 µm in primary and 56 µm in secondary in wild-type follicles. In *Rspo2*^*Tg/Tg*^ ovaries, primary follicles contain oocytes of 55 µm in diameter, corresponding to the diameter of oocytes of secondary wild-type follicles (Fig. 4a). In addition, 11% of the follicles are abnormal primordial follicles surrounded by a layer of flattened granulosa cells, with oocytes of 63 µm in diameter, corresponding to the size of the oocyte in tertiary/antral wild-type follicles. At 21d, 82% of the follicles are primordial and primary follicles in *Rspo2*^*Tg/Tg*^ ovaries. Our data demonstrate that *Rspo2* ablation does not impair oocyte growth despite the growth arrest of the primary follicles.

### Oocyte-secreted RSPO2 activates WNT/CTNNB1 signaling in granulosa cells

The phenotype of *Rspo2*^*Tg/Tg*^ ovaries shows similarities with *Gdf9*^*−/*^^−^ ovaries regarding the lack of granulosa cell proliferation and the maintenance of the oocyte growth [12, 13]. This prompted us to investigate the expression level of key genes involved in GDF9 signaling like *Gdf9* and its receptors *Bmpr2* and *Alk4* [41] by qRT-PCR analyses. None of these transcripts were downregulated, suggesting that RSPO2 signaling acts independently of the GDF9 pathway (Fig. 4b). In addition, we analyzed the expression of *Kit* and *KitL*, two factors involved in primordial follicle activation [42]. *Kit* expression was similar in wild-type and mutant ovaries, indicating the maintenance of this signaling pathway from the oocyte toward granulosa cells. The expression of *KitL* was lowered in *Rspo2*^*Tg/Tg*^ ovaries at 12d, but the expression of the granulosa cell marker *Foxl2* was also reduced, suggesting that *KitL* reduction is associated with the diminution of the granulosa cell numbers.

**Fig. 5:**
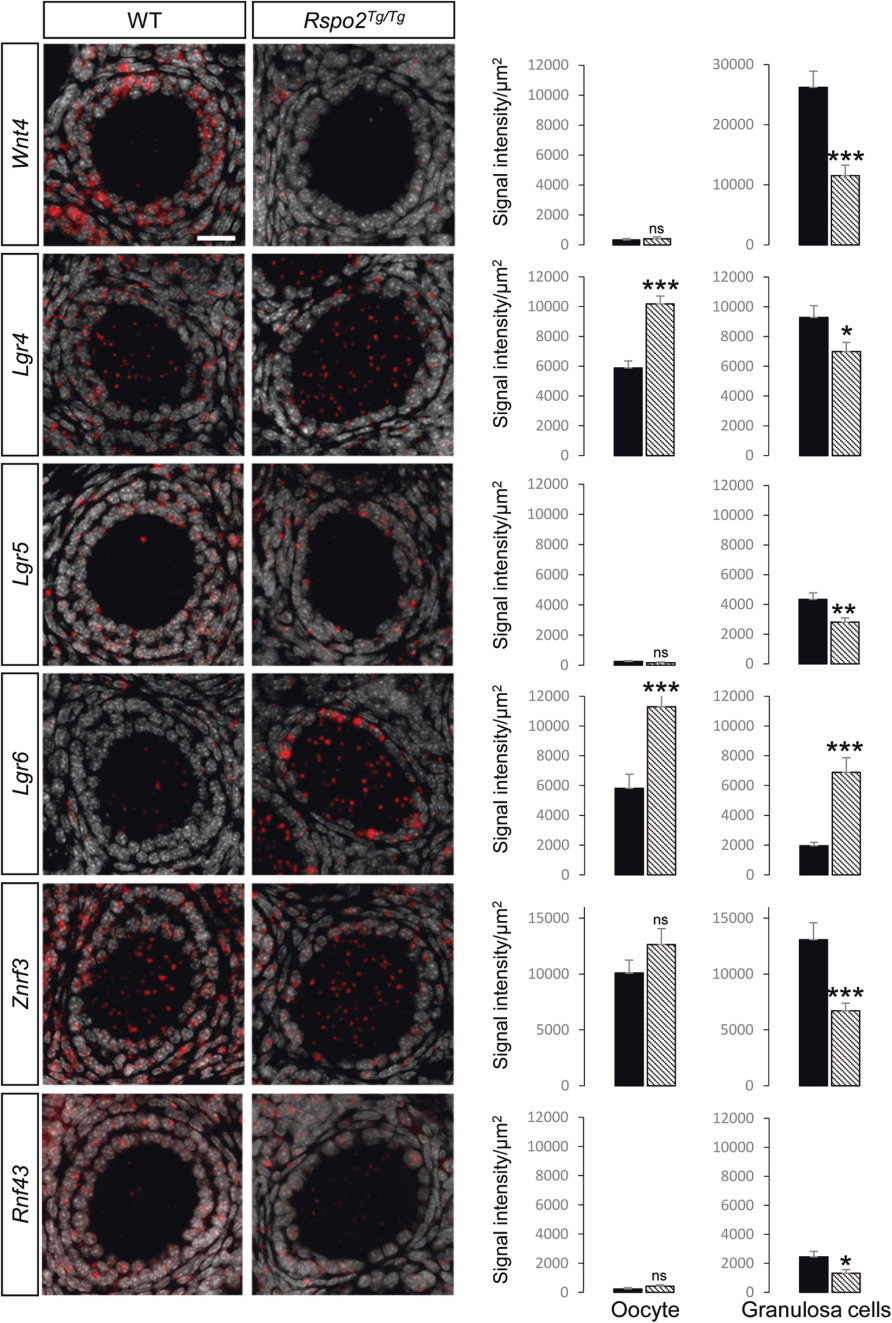
RSPO2 activates WNT/CTNNB1 signaling in granulosa cells. In situ hybridization analysis of six main actors of the RSPO/WNT/CTNNB1 signaling pathway, e.g., *Wnt4*, *Lgr4, Lgr5, Lgr6, Znrf3,* and *Rnf43* and expression signal quantification in wild-type (WT) and *Rspo2*^*Tg/Tg*^ follicles at 12d. The number of counted follicles (of three ovaries per genotype) is *n* = 20, 11, 14, 17, 15, and 6 for WT and *n* = 26, 15, 14, 23, 16, and 9 for *Rspo2*^*Tg/Tg*^ ovaries in *Wnt4*, *Lgr4, Lgr5, Lgr6, Znrf3,* and *Rnf43* staining, respectively. In WT follicles, *Lgr5, Rnf43,* and *Wnt4* expression is restricted to the granulosa cells, whereas *Lgr4*, *Lgr6*, and *Znrf3* are expressed in oocytes and granulosa cells. In *Rspo2*^*Tg/Tg*^ granulosa cells, the expression of all these markers, except *Lgr6*, is downregulated compared with WT. Data are presented as mean ± SEM. Student’s *t* test, unpaired two sided (*p < 0.05; **p < 0.01; ***p < 0.001).

RSPO2 is an activator of WNT/CTNNB1 signaling that is mediated by nuclear translocation of CTNNB1. Immunolocalization analyses of CTNNB1 using an antibody that recognizes nuclear and cytoplasmic, including membrane forms, of CTNNB1 revealed an overall downregulation of CTNNB1 in granulosa cells (Fig. 4c). Quantification analyses using DAPI to measure CTNNB1 expression restricted to nuclei revealed higher levels of CTNNB1 in the nuclei of granulosa cells in wild types when compared with *Rspo2*^*Tg/Tg*^ follicles (Fig. 4d). Our data suggest that RSPO2 promotes nuclear translocation of CTNNB1. To verify the activation of this signaling pathway, we then studied the level of expression of different genes including *Axin2*, a universal readout of the activation of this signaling pathway [43]. In situhybridization revealed that *Axin2* is highly expressed in the oocyte and weaker in granulosa cells in wild-type ovaries at 12d (Fig. 4e). In *Rspo2*^*Tg/Tg*^ follicles, the expression of *Axin2* is restricted to the oocytes. Quantification of the signal intensity confirmed the downregulation of *Axin2* in granulosa cells as evidenced by a robust decrease of *Axin2* mRNA in these cells (Fig. 4f). Next, we performed expression analyses for the main actors of the RSPO/WNT/CTNNB1 signaling pathway, e.g., the receptors *Lgr4*, *Lgr5*, and *Lgr6* and the two E3-ubiquitin ligases *Znrf3* and *Rnf43*. Among them, *Lgr5, Rnf43,* and *Znrf3* have been described to be targets of WNT/CTNNB1 signaling [17, 44]. In wild-types, *Lgr5* and *Rnf43* expression is restricted to the granulosa cells, whereas *Lgr4*, *Lgr6,* and *Znrf3* are expressed in oocytes and granulosa cells (Fig. 5). In *Rspo2*^*Tg/Tg*^ granulosa cells, *Lgr5, Znrf3,* and *Rnf43* expression is downregulated, confirming that RSPO2 is involved in the activation of WNT/CTNNB1 signaling in early developing follicles.

It is noteworthy that WNT4 can signal via the WNT/CTNNB1 pathway in granulosa cells and promote late follicular development [45]. In the *Rspo2*^*Tg/Tg*^ ovaries, we observed a downregulation of *Wnt4* that likely contributes to the proliferation defects of the granulosa cells in primary follicles (Fig. 5). Altogether, our data suggest that RSPO2 activates WNT/CTNNB1 signaling in granulosa cells.

### WNT/CTNNB1 signaling is required for granulosa cell proliferation

**Fig. 6:**
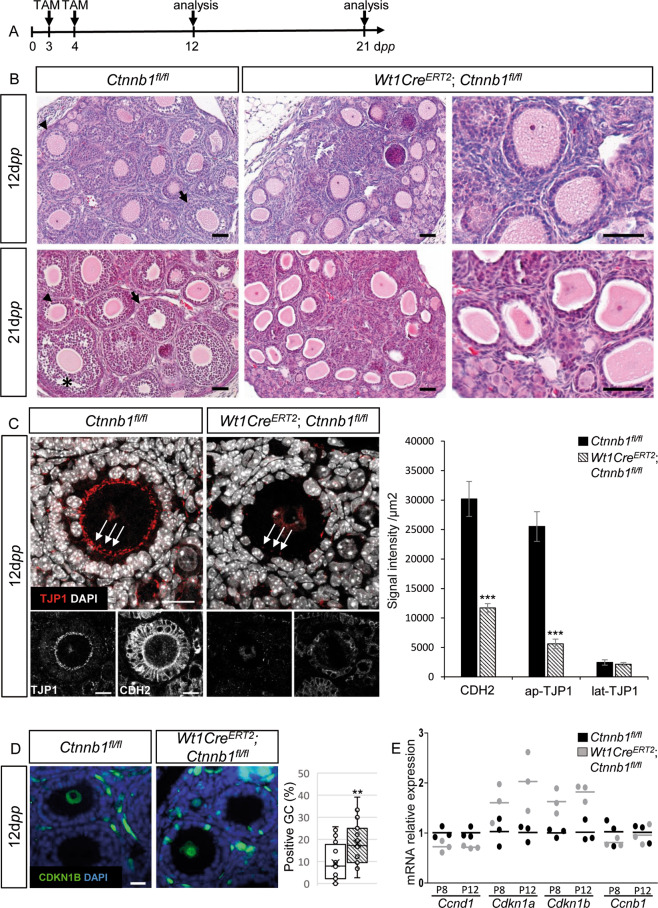
Genetic deletion of *Ctnnb1* in granulosa cells induces similar phenotypes as *Rspo2*^*Tg/Tg*^ ovaries. **a** Timeline of tamoxifen administration (TAM) at 3 and 4 d*pp* and ovary analyses of control (*Ctnnb1*^*fl/fl*^) and mutant (*Wt1Cre*^*ERT2*^;* Ctnnb1*^*fl/fl*^) mice at 12 and 21 d*pp*. **b** Histological section analyses (HE stainings) of *Ctnnb1*^*fl/fl*^ and *Wt1Cre*^*ERT2*^; *Ctnnb1*^*fl/fl*^ ovaries at 12 and 21 d*pp*. Mutant ovaries lack secondary (arrowheads), tertiary (arrows), and antral (*) follicles in contrast to *Ctnnb1*^*fl/fl*^ ovaries. Scale bars, 50 µm. **c** Immunodetection and quantitative analyses of TJP1 (tight junctions) and CDH2 (adherens junctions) in *Ctnnb1*^*fl/fl*^ and *Wt1Cre*^*ERT2*^; *Ctnnb1*^*fl/fl*^ ovaries at 12 d*pp*. CDH2 and TJP1 signals are decreased in *Wt1Cre*^*ERT2*^; *Ctnnb1*^*fl/fl*^ follicles, notably at the interface between the oocyte and granulosa cells (indicated by white arrows). Data are presented as mean ± SEM. *n* = 6 *Ctnnb1*^*fl/fl*^ and *n* = 7 *Wt1Cre*^*ERT2*^; *Ctnnb1*^*fl/fl*^ follicles of each genotype. Student’s *t* test, unpaired two sided (***p < 0.001). Scale bars, 20 µm. **d** Immunodetection and quantitative analyses of CDKN1B (p27) to assess the proliferation status of granulosa cells in *Ctnnb1*^*fl/fl*^ and *Wt1Cre*^*ERT2*^; *Ctnnb1*^*fl/fl*^ ovaries at 12 d*pp* (*n* = 700 and 499 granulosa cells from 18 and 16 follicles, respectively, of one transplanted ovary per genotype). Data are presented in a box and whisker plot representation to illustrate positive granulosa cell dispersion according to the follicle considered. Student’s *t* test, unpaired two sided (*p < 0.05; **p < 0.01). Scale bar, 20 µm. **E** QRT-PCR analysis of, *Ccnd1* (CyclinD1), *Cdkn1a* (p21), *Cdkn1b* (p27), and *Ccnb1* (CyclinB1). Data are presented as individual data points. *n* = 3 individual ovary per genotype. Mean values are indicated as black (*Ctnnb1*^*fl/fl*^) and gray (*Wt1Cre*^*ERT2*^; *Ctnnb1*^*fl/fl*^) bars.

To assess the contribution of WNT/CTNNB1 signaling in granulosa cell proliferation, conditional deletion of *Ctnnb1* was performed by using *Wt1Cre*^*ERT2*^; *Ctnnb1*^*fl/fl*^ mice. *Wt1Cre*^*ERT2*^ is driven by the endogenous *Wt1* promoter and is expressed in granulosa cells [46]. Genetic deletion was induced by tamoxifen administration at 3 and 4 d*pp* and analyses were performed at 12 and 21 d*pp* (Fig. 6a). At both stages, mutant ovaries lack secondary follicles (Fig. 6b), as previously observed in *Rspo2*^*Tg/Tg*^ transplanted ovaries (Fig. 1b). FOXL2 and AMH immunodetection analyses revealed that granulosa cells express low levels of AMH in *Wt1Cre*^*ERT2*^;* Ctnnb1*^*fl/fl*^ ovaries at 12 d*pp*, as observed in *Rspo2*^*Tg/Tg*^ ovaries (Fig. 2a).

Next, the analysis of the actors of the WNT/CTNNB1 signaling pathway showed that the active nuclear form of CTNNB1 appears to be less abundant in *Wt1Cre*^*ERT2*^;* Ctnnb1*^*fl/fl*^ ovaries in comparison with wild types (Fig. S4a, b). In addition, in situ hybridization experiments at 21 d*pp* showed that *Axin2* expression is reduced in *Wt1Cre*^*ERT2*^;* Ctnnb1*^*fl/fl*^ granulosa cells in comparison with *Ctnnb1*^*fl/fl*^ ovaries (Fig. S4c, d). This indicates that WNT/CTNNB1 signaling is activated in granulosa cells in early developing follicles and RSPO2 is a/the main factor of this activation. Furthermore, the adherens and tight junction markers CDH2 and TJP1, respectively, are less abundant in *Wt1Cre*^*ERT2*^;* Ctnnb1*^*fl/fl*^ follicles than in *Ctnnb1*^*fl/fl*^ follicles at 12 dpp (Fig. 6c). This suggests that granulosa cells are not packed enough to allow the formation of adherens and tight junctions in ovaries lacking *Ctnnb1*.

We then characterized the proliferative status of the granulosa cells. QRT-PCR experiments revealed that the direct target of CTNNB1, *Ccnd1*, is downregulated in mutant ovaries, whereas the two cell cycle inhibitors *Cdkn1a* and *Cdkn1b* are upregulated (Fig. 6e). Moreover, immunolocalization of CDKN1B showed an upregulation of this marker in granulosa cells in mutant ovaries (Fig. 6d), highlighting an arrest in the G1 phase of the cell cycle, as evidenced in *Rspo2*^*Tg/Tg*^ ovaries (Fig. 3b). QRT-PCR analysis indicated an absence of significant modification of the expression of *Gdf9* and its receptors *Bmpr2* and *Alk4* (Fig. S4e). In contrast, *KitL* expression was decreased at 8 and 12 d*pp*, whereas oocyte-specific *Kit* expression was increased in the *Wt1Cre*^*ERT2*^;* Ctnnb1*^*fl/fl*^ ovaries (Fig. S4e). In summary, conditional deletion of *Ctnnb1* within the granulosa cells of primordial follicles impaired their proliferation and prevented follicular growth beyond the primary follicular stage in a GDF9 signaling independent manner. Given that this phenotype recapitulates the phenotype induced by *Rspo2* ablation, our data confirm that RSPO2 is an enhancer of WNT/CTNNB1 signaling in granulosa cells. Remarkably, our data show that RSPO2/CTNNB1 is mediated by paracrine signaling with the oocyte-secreted RSPO2 promoting CTNNB1 activation in the neighboring granulosa cells.

## Discussion

In the ovary, the regulation of *Rspo2* expression is attributed to the oocyte-specific homeobox gene, *Nobox* as evidenced by microarray analysis of *Nobox* loss-of-function ovaries showing a downregulation of *Rspo2* [47] and by in silico analysis of the *Rspo2* promoter revealing the presence of two NOBOX binding elements [48]. However, the ablation of *Nobox* triggers rapid degeneration of perinatal oocytes, thus impacting the expression of many oocyte-specific genes like *Rspo2* [49]. The expression of *RSPO2* has also been shown to be regulated by the transcription factor FOXL2 in goat ovaries [50]. In mouse, FOXL2 cannot directly regulate *Rspo2* given that *Rspo2* is expressed in the oocyte and *Foxl2* in granulosa cells. Thus, regulation of *Rspo2* expression in the postnatal oocyte remains to be investigated.

**Fig. 7:**
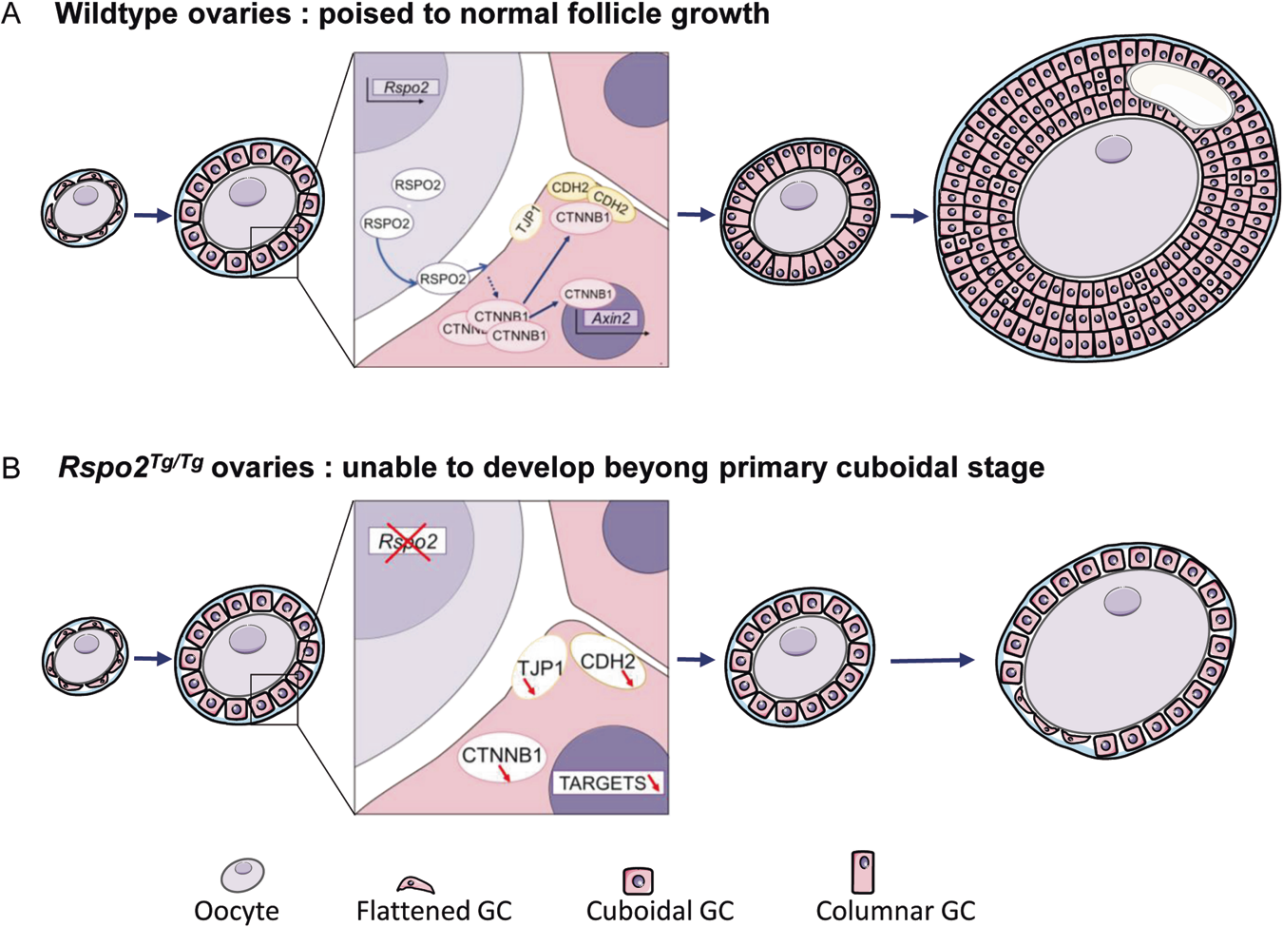
Proposed model for RSPO2 signaling in mouse ovaries. **a** Follicular growth requires the oocyte-secreted RSPO2 protein. RSPO2 promotes the stabilization of CTNNB1 in the neighboring granulosa cells and, in turn, their proliferation and cell adhesion. This leads to follicular growth and development of antral follicles. **b** In the absence of RSPO2, activation of CTNNB1 is decreased in granulosa cells, thus leading to defects in proliferation and adhesion. Although oocyte growth occurs as in control follicles, the *Rspo2*^*Tg/Tg*^ follicles remain as primary follicles containing flattened and cuboidal granulosa cells.

Our study exemplifies the importance of the communication between the oocyte and the neighboring granulosa cells. Here we show that the oocyte factor RSPO2 induces the activation of WNT/CTNNB1 signaling, also called canonical WNT signaling, in granulosa cells, thereby promoting cell cycle progression and eventually follicular growth (Fig. 7). Consequently, ablation of *Rspo2* leads to a lack of growing numbers of granulosa cells that are required to develop secondary follicles and follicles remain at the primary stage. This arrest is associated with changes of the granulosa cells when they age as evidenced by downregulation of AMH. This does not trigger follicular degeneration, since primary follicles are still observed in 2-month-old *Rspo2*^*Tg/Tg*^ ovaries. Thus, these follicles unlikely gain the competency to become atretic as previously reported in *Gdf9*^*−/*^^*−*^ ovaries [12]. Overall, our results demonstrate that RSPO2 is an oocyte-secreted mitogen required for granulosa cell proliferation and eventually differentiation.

In *Foxl2* loss-of-function mice, granulosa cells hardly proliferate and this is associated with their inability to become cuboidal cells [36]. In agreement with this, previous results showed that the change in shape from flattened to cuboidal granulosa cells is associated with an increase in proliferation [51, 52]. Indeed, the flat granulosa cells occasionally start to proliferate in response to unknown signals in primordial follicles [8]. The two adjacent daughter granulosa cells exhibit the first substantial region of adhesion that extends perpendicular to the oocyte surface in primordial follicles. The dividing granulosa cells become more packed on the oocyte surface and then cuboidal due to the space constraints. In *Rspo2*^*Tg/Tg*^ follicles, KIT signaling is not impaired at an earlier stage like 8d, suggesting that the activation of the primordial follicles is maintained as previously described by [4]. The granulosa cells proliferate less than in wild types and exhibit less adherens and tight junctions. Some granulosa cells develop until the cuboidal stage but rarely evolve into dense polarized columnar cells in comparison with wild types. It is likely that the lower number of granulosa cells prevents them from forming contacts between them and between them and the oocyte and this might hinder the formation of junctions in *Rspo2*^*Tg/Tg*^ follicles and their cuboidalization. Interestingly, it has been shown that the elimination of protein geranylgeranylation, involved in posttranslational modifications of proteins, impairs cell adhesion between the oocyte and the granulosa cells and promotes the arrest at the primary–secondary follicle transition [53]. Thus, the decrease of tight and adherens junctions in *Rspo2*^*Tg/Tg*^ and *Wt1Cre*^*ERT2*^;* Ctnnb1*^*fl/fl*^ ovaries might contribute to the blockage of growth of primary follicles.

The oocyte factor GDF9 plays a central role in the signaling from the oocyte to the somatic cells [6]. Strikingly, GDF9 and RSPO2 signaling are both involved in granulosa cell proliferation. RSPO2 activates WNT/CTNNB1 signaling and GDF9 signaling acts through the BMPR2/ALK4 heterodimer receptor and SMAD2/3 pathway in the granulosa cells [41]. Recently, GDF9 was also reported to promote the formation of the transzonal projections from the neighboring granulosa cells to the oocyte through the zona pellucida [11]. The mechanism of action of RSPO is still not very well understood, but RSPOs might use filopodia to reach their target cells as has been shown for WNT ligands [54]. The lack of filopodia in *Gdf9*^*−/*^^−^ follicles may prevent the delivery of RSPO2 to the granulosa cells once the zona pellucida is formed, thus preventing the follicular growth. GDF9 might therefore promote RSPO2/WNT signaling in the ovarian follicle.

Considering the fertility defect in heterozygous *Rspo2* mutant mice, the severe follicular growth defect in *Rspo2* homozygous mutant ovaries and the conservation of RSPO2 expression in ovaries in a little girl, we can assume that *RSPO2* is a candidate gene for premature ovarian insufficiency (POI) in women. Bouilly et al. [48] previously screened 100 patients suffering from POI and did not associate RSPO2 as a potential cause. This might be due to the heterozygous condition. Indeed, homozygous mutations of RSPO2 have been described in human patients with a rare malformation disorder, the tetra-amelia and lung hypo/aplasia syndrome characterized by the absence of the forelimbs and pulmonary anomalies [55, 56]. The patients die at birth due to respiratory distress but in most cases, ultrasonography evaluation leads to an early termination of the pregnancy. Few cases have been described and the pathological examination of one female fetus at the 22nd gestational weeks excluded genitourinary anomalies, but there is no detail on folliculogenesis [57]. At this age, primary follicles are present, but secondary follicles are still rare, thus preventing to know whether loss of *RSPO2* could induce a follicular growth defect in these patients. Given the severity of the phenotype described here, the different actors involved in RSPO2/WNT signaling in the ovary, once identified, will likely become new candidates for the diagnosis of POI.

## Material and methods

### Mouse strains and genotyping

The experiments were carried out in compliance with the relevant institutional and French animal welfare laws, guidelines, and policies and have been approved by the French ethics committees (CIEPAL, accreditation APAFIS#4035–2016012712367693). The mice were kept on a mixed 129/C57Bl6/J genetic background. Homozygous mutant *Rspo2*^*Tg/Tg*^ embryos were identified by limb malformations and hypoplasia and confirmed by genotyping as described in [29, 35]. Homozygous females carrying the *Ctnnb1*^*fl*^ allele were mated with *Wt1Cre*^*ERT2*^ males to generate *Wt1Cre*^*ERT2*^; *Ctnnb1*^*fl/fl*^ mice [58, 59]. Activation of the Cre recombinase was performed by intraperitoneal injections of 0.4 mg of tamoxifen (Sigma-Aldrich) dissolved in corn oil (Sigma-Aldrich) per pup. All the pups of the litters were treated in the same way, and genotypes were determined when samples were collected. Hence, the controls (*Ctnnb1*^*fl/fl*^) got the same treatments as *Wt1Cre*^*ERT2*^; *Ctnnb1*^*fl/fl*^ mutants.

### Transplantation of fetal mouse ovaries

Wild-type and *Rspo2*^*Tg/Tg*^ embryos were collected from 18.5 d*pc* fetuses. Ovaries were transplanted under the kidney capsule of 6-week-old female athymic mice (Envigo, France). Transplants were dissected from host animals 8, 12, 21 days or 2 months posttransplantation and processed as described below.

### Histological analysis

Kidneys including the transplants or ovaries were dissected, fixed in Bouin’s solution overnight embedded in paraffin, sectioned at 5 µm thickness, and stained with hematoxylin and eosin (HE). Pictures were taken with an Axioscope 2 (Zeiss) or MZ9.5 (Leica) microscope coupled with an Axiocam MRc5 (Zeiss) or DHC490 (Leica) camera and Axiovision 4.8 (Zeiss) or application suite V3.3.0 (Leica) software, and processed with Adobe Photoshop for mosaics and Fiji [60].

### Morphometric analyses of ovarian follicles

The developmental stages of ovarian follicles (primordial, primary, secondary, tertiary, and antral) were determined on HE stained Bouin’s fixed sections, on the basis of the standards established by [61]. Follicles were counted in ten randomly selected sections of three wild-type and mutant grafted ovaries and expressed as a percentage of the total number of follicles counted. The average oocyte and follicle diameters were evaluated as performed by [42]. Only the follicles sectioned through the nucleus of the oocyte were scored.

### In situ hybridization analyses

Samples were fixed in 4% paraformaldehyde (PFA) overnight at room temperature. 5 µm sections were processed for RNA in situ detection using the RNAscope 2.0 High Definition-RED Kit according to the manufacturer’s instructions (ACDBio, [62]). *Axin2, Lgr4, Lgr5, Lgr6, Rnf43, Rspo2, Wnt4*, and *Znrf3* probes were designed by ACDBio. Slides were counterstained with DAPI diluted in the mounting medium at 10 µg/ml (Vectashield, Vector laboratories) to detect nuclei. RNAscope results were examined under a LSM 780 NLO inverted Axio Observer.Z1 confocal microscope (Carl Zeiss Microscopy GmbH, Jena, Germany) using a C-Apo 40X water 1.2 NA objective. The lasers used were diodes laser (405, 488, 532, and 635 nm). The microscope was equipped with a galvanometric stage in order to do z acquisitions. For *Rspo2* detection, slides were counterstained with 50% Hematoxylin for 10 s and incubated in 0.1% sodium bicarbonate for 1 min. Slides were then mounted in VectaMount AQ medium (Vector Lab), scanned with Vectra Polaris Automated Imaging System, and examined with Phenochart Software (PerkinElmer).

### Immunolabeling analyses

Mouse samples were fixed in 4% PFA overnight at 4 °C and then processed for paraffin embedding. Microtome sections of 6 μm thickness were processed for immunostaining, and antigens were retrieved in Envision Flex Target Retrieval solution (pH9) on the PTlink Pre Treatment Module (DAKO, Agilent). The following dilutions of primary antibodies were used: AMH (C-20, sc6886, Santa Cruz) 1:200, CDH2 (cat 33–3900, Invitrogen) 1:200, CTNNB1 (cat 2206, Sigma) 1:100, FOXL2 (NB100-1277, Novus Bio) 1:400, LAMA1 (cat L9393, Sigma) 1:150, MKi67 (clone SP6, cat 9106, Thermo-Scientific) 1:200, p27 (sc-528, Santa Cruz) 1:200, PHH3 (ab14955, Abcam) 1:300, SF1 (kindly provided by Prof. Morohashi) 1:1000, TJP1 (cat 40–2300, Invitrogen) 1:200. Slides were counterstained with DAPI diluted in the mounting medium (Vectashield, Vector laboratories). Imaging was performed with a motorized Axio ImagerZ1 microscope (Zeiss) coupled with an Axiocam Mrm camera (Zeiss) and processed with Axiovision LE (Zeiss) or on a LSM 780 NLO inverted Axio Observer.Z1 confocal microscope (Carl Zeiss Microscopy GmbH, Jena, Germany) using a C-Apo 40X water 1.2 NA objective when quantification was needed. Images were acquired in mono-photon mode using a LASER diode 405 nm and/or Argon LASER (458, 488, 514 nm) nm and/or DPSS 561 nm and/or HeNe 633 nm. Fluorescence emission was detected on a descanned spectral GaAsp PMT 32 channels. The microscope z-drive was used for z acquisitions.

Formalin-fixed paraffin-embedded ovaries from girls aged 12 postnatal months dying suddenly and unexpectedly were accrued under the French autopsy law that allows the use of such tissues for in-depth anatomopathological examination (Law 94–654 published on July 29, 1994). Autopsy was performed with informed consent of the parents in all cases. These methods were carried out in accordance with relevant guidelines and regulations. All the experiments and experimental protocols on human subjects were approved by the institutional committee of the French agency for biomedical research (Agence de la Biomédecine, Saint-Denis la Plaine, France). Immunohistochemistry was performed as previously described (François et al., Scientific report 2017). Slides were incubated overnight with primary antibodies for RSPO2 (Dilution 1/100; Sigma, HPA024764). They were counterstained in hematoxylin, dehydrated and mounted in Eukitt (Sigma).

### Image posttreatments and quantitative analyses

Images were assembled using the open source software platform OMERO (https://www.openmicroscopy.org/omero/). Posttreatment image acquisition and analysis were performed on Fiji [60], using a homemade semiautomated macro when needed.

For CTNNB1 expression quantification, z-stack images were processed through sum slices projection. Nuclear CTNNB1 signal intensity was determined as the signal colocalized with DAPI staining and cytoplasmic CTNNB1 signal intensity was determined as the signal localized outside the DAPI staining but within granulosa cell crown (drawn manually). CTNNB1 signal intensity was quantified as raw integrated density after threshold adjustment normalized on nuclei or cytoplasmic area (µm^2^). *Axin2*, TJP1, and CDH2 expression was quantified on confocal z-stack images according to the different steps described in Fig. S5. For all the three *Lgr, Rnf43, Wnt4*, and *Znrf3*, signal intensity was quantified on z-stack images processed through sum slices projection. Oocyte surface was obtained by manual drawing of the external contour of the oocyte and granulosa cell layer region of interest (ROI) was determined as the whole follicle area (drawn manually) minus the oocyte surface on DAPI channel. Signal intensity within the oocyte and the granulosa cell ROI was quantified as raw integrated density after threshold adjustment normalized on oocyte and granulosa cell surface (µm^2^), respectively.

Cell proliferation was assessed by counting MKI67, PHH3, and CDKN1B-positive cells among total DAPI-positive granulosa cells on at least ten different follicles per genotype and per marker.

### Quantitative PCR analysis

RNA from snap frozen ovaries was extracted using the RNeasy Micro Kit (Qiagen) and reverse transcribed using the RNA RT-PCR kit (Promega). Primers (Supplementary Table 1) were designed by Roche Assay Design Center (http://qpcr.probefinder.com/organism.jsp). All real-time PCR assays were carried out using LightCycler 480 SYBR Green Master kit (Roche). QRT-PCR were performed on cDNA from one gonad and compared with a standard curve. Relative expression levels of each gene were determined in the same run and normalized on the levels of endogenous *Sdha* cDNA. QPCR were repeated in duplicate on cDNA issued from three ovaries of each genotype.

### Statistical analysis

When appropriate, values are depicted as mean ± SEM. Data were analyzed by unpaired two-sided Student’s *t* test using Microsoft Excel. Asterisks highlight the pertinent comparisons and indicate levels of significance: *p < 0.05, **p < 0.01, and ***p < 0.001. Cell proliferation counting is shown as box and whisker plot representation to illustrate positive granulosa cell dispersion according to the follicle considered. QRT-PCR analyses are presented as individual data points. The mean value between individual points is shown as a horizontal bar.

## Supplementary information

Sup. Figure S1

Sup. Figure S2

Sup. Figure S3

Sup. Figure S4

Sup. Figure S5

Sup. Figure Table 1

Supplementary Figures Legends
