# Supplementary figures and images for: R-spondin2 signaling is required for oocyte-driven intercellular communication and follicular growth
Source: Cell Death Differ. 2020 Apr 27;27(10):2856–71. doi: 10.1038/s41418-020-0547-7 (PMC7493947; doi:10.1038/s41418-020-0547-7)

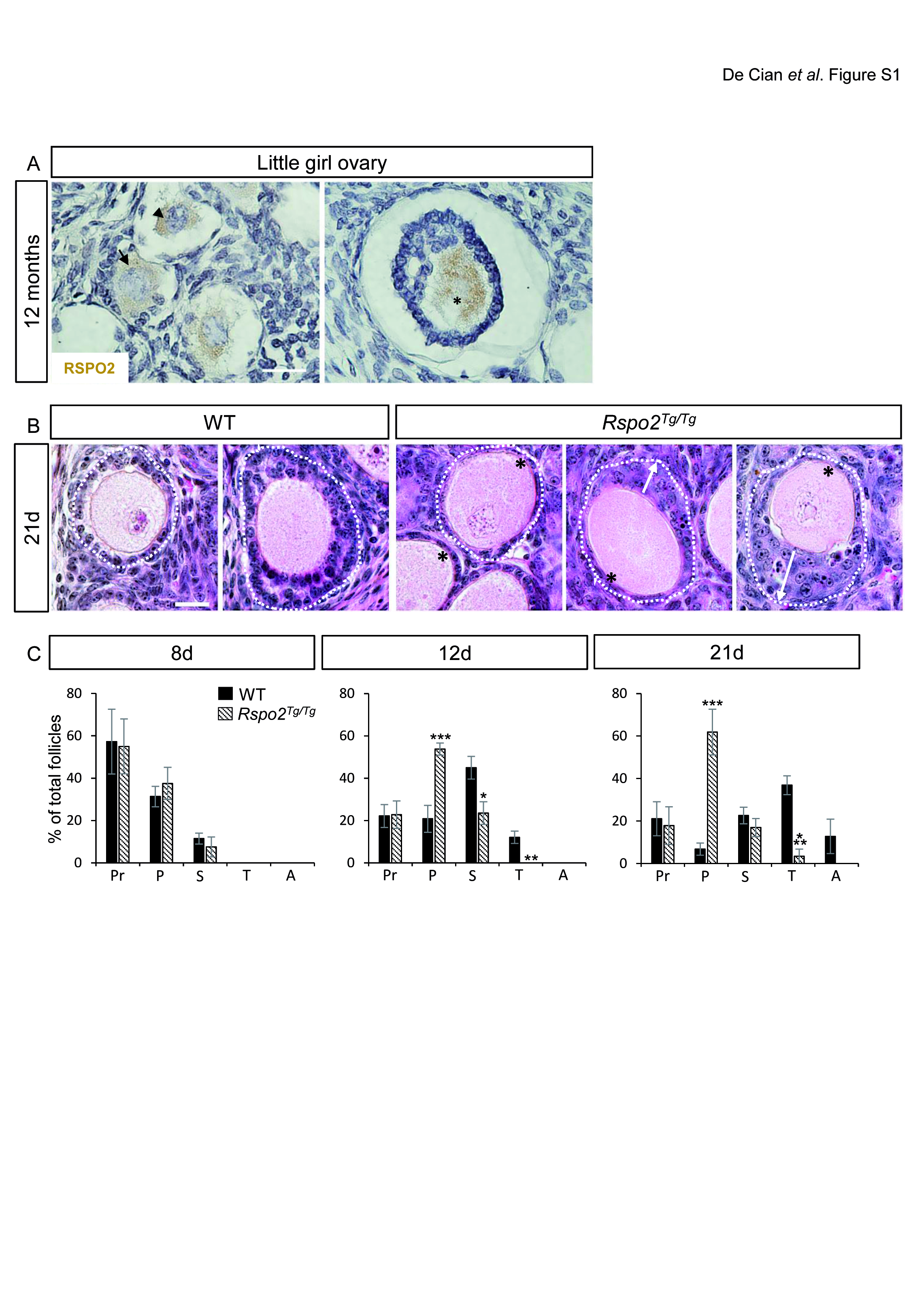

Supplement: Supplementary file 1 — Sup. Figure S1 [file 41418_2020_547_MOESM1_ESM.tif]

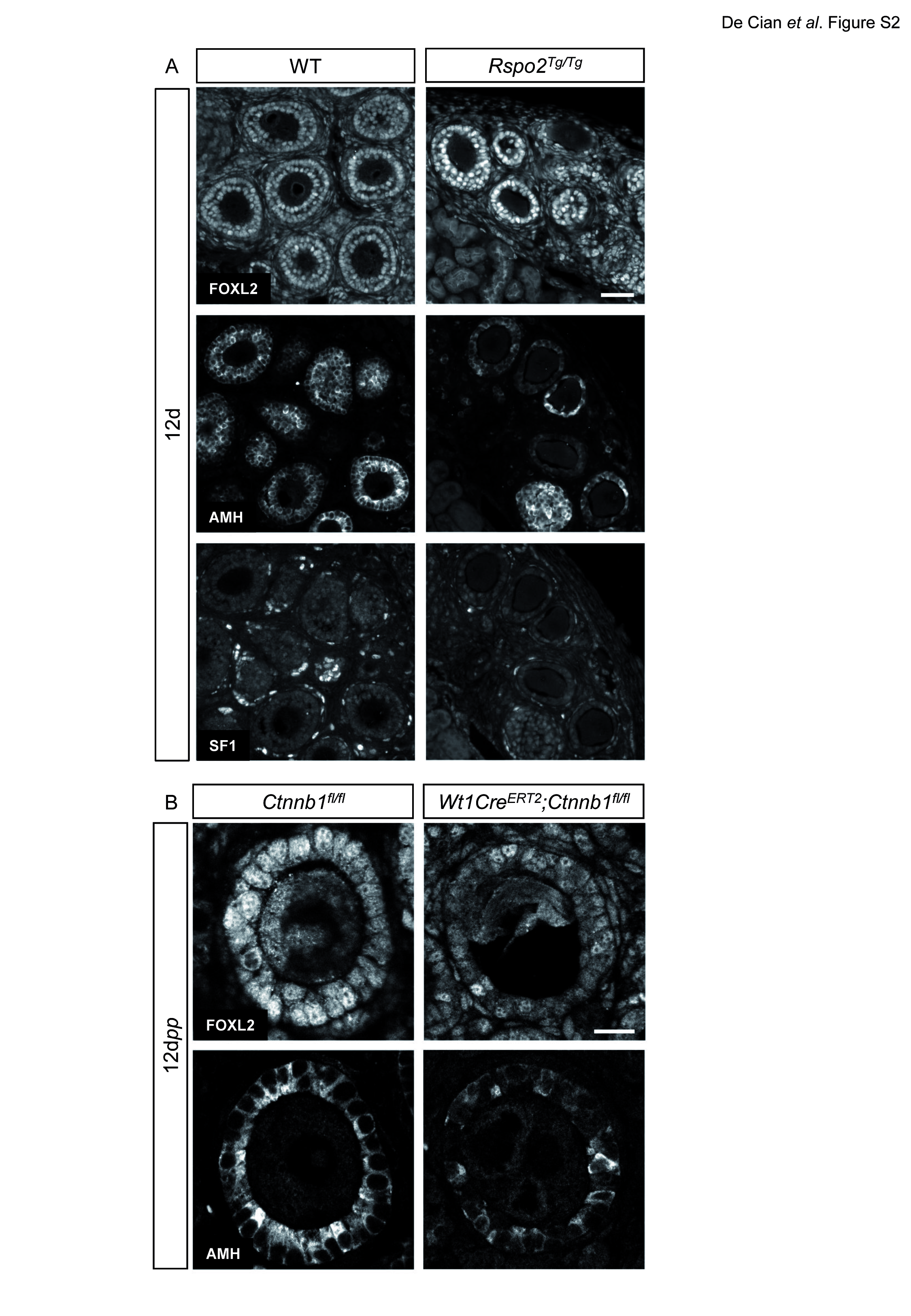

Supplement: Supplementary file 2 — Sup. Figure S2 [file 41418_2020_547_MOESM2_ESM.tif]

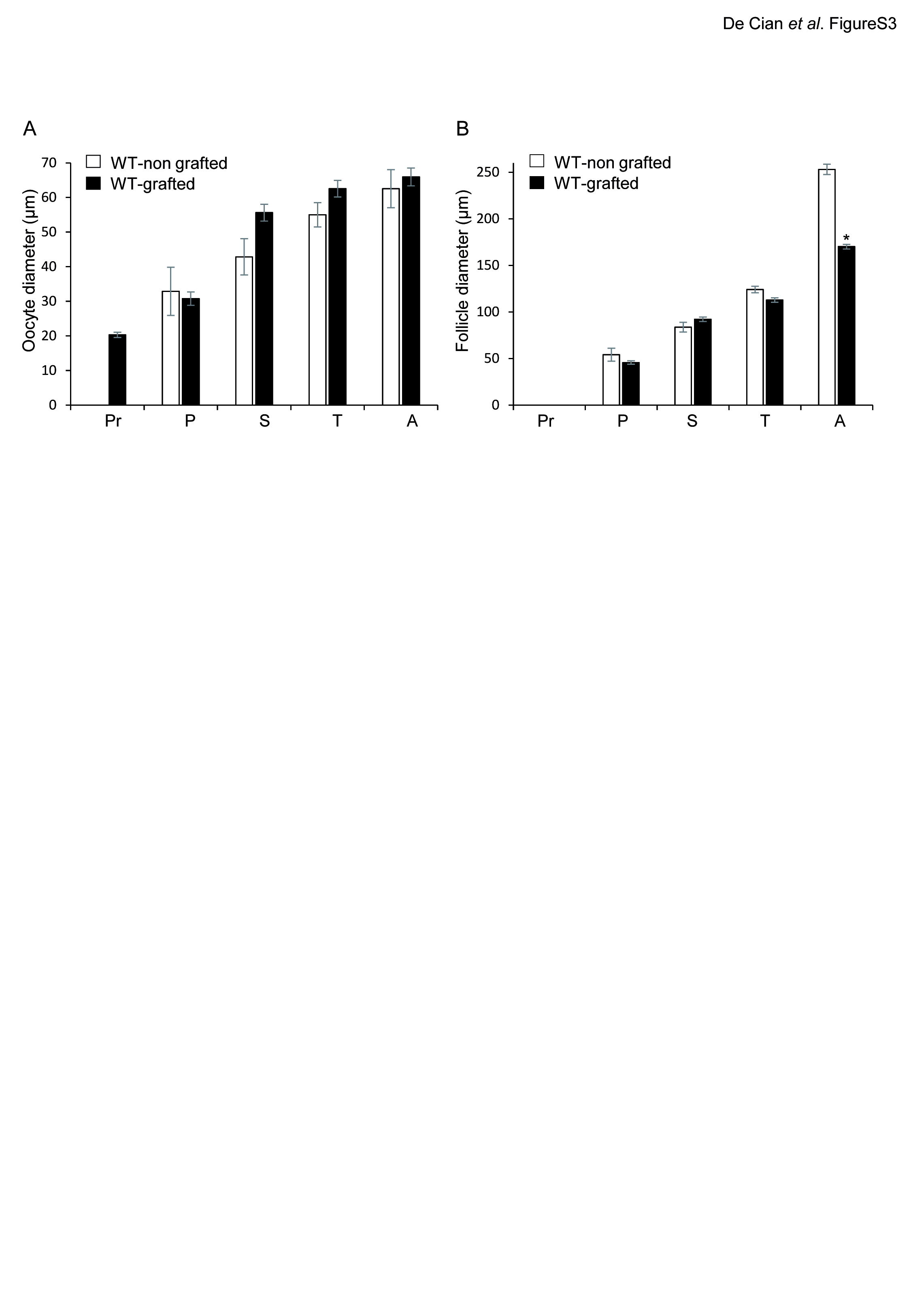

Supplement: Supplementary file 3 — Sup. Figure S3 [file 41418_2020_547_MOESM3_ESM.tif]

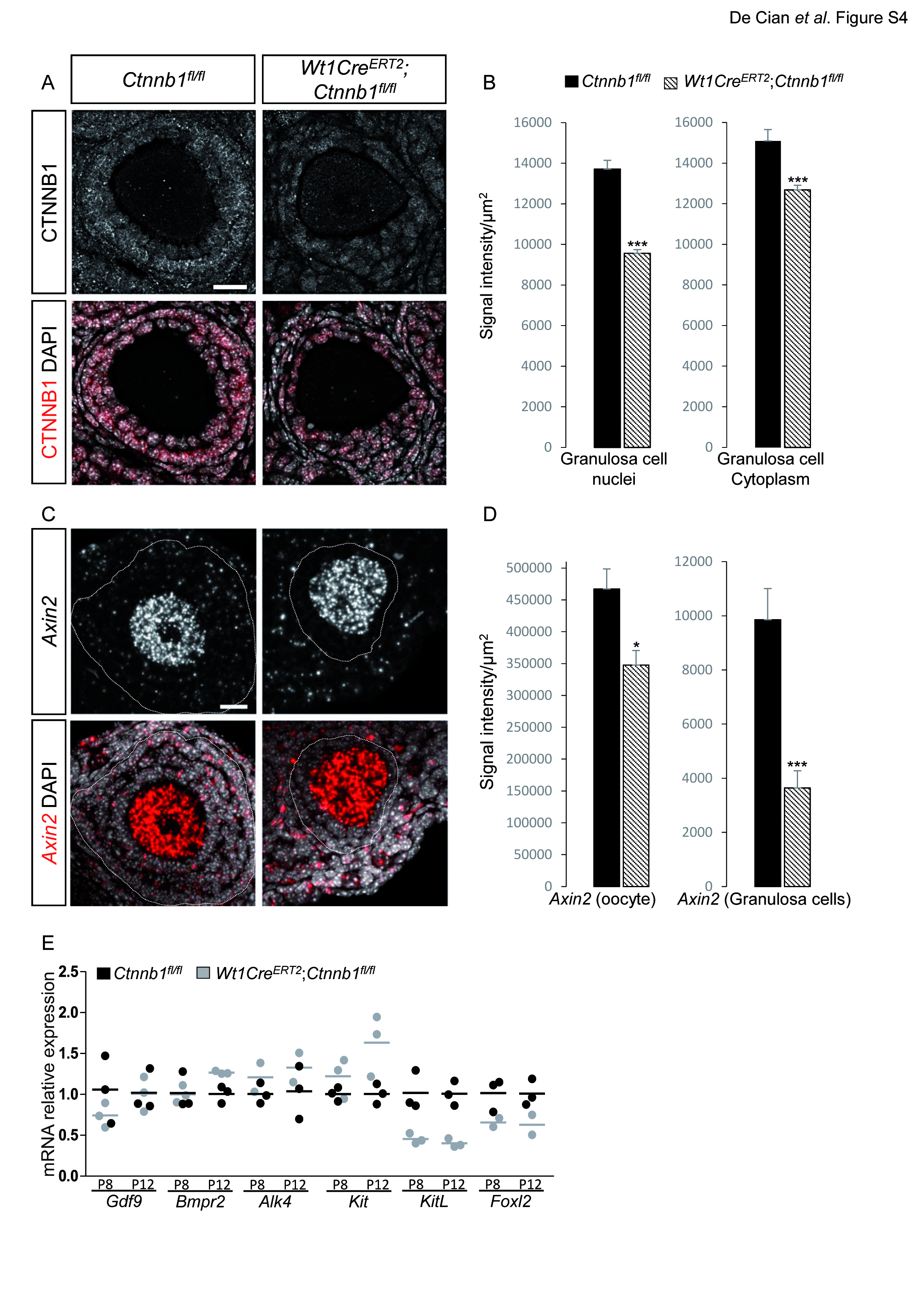

Supplement: Supplementary file 4 — Sup. Figure S4 [file 41418_2020_547_MOESM4_ESM.tif]

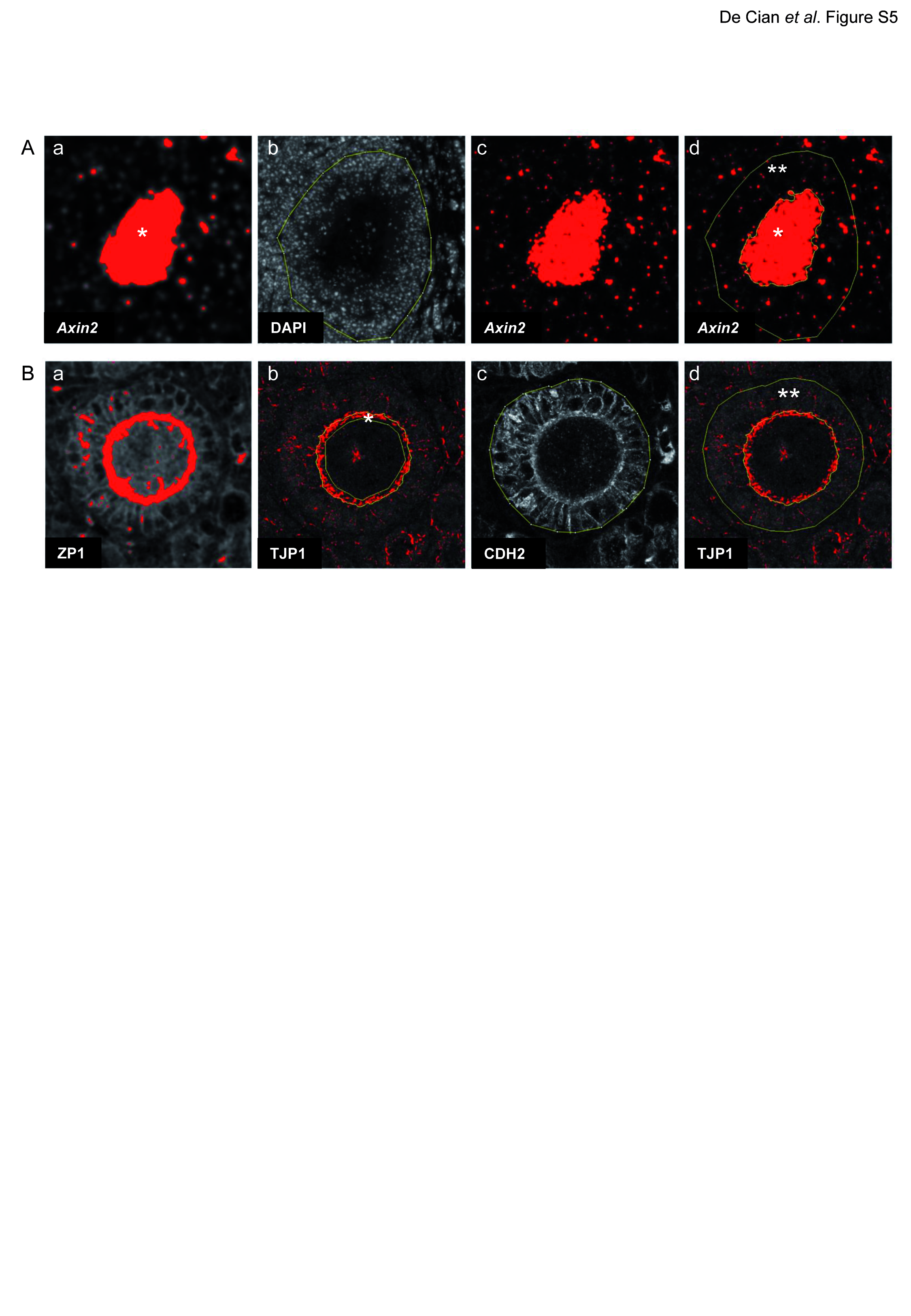

Supplement: Supplementary file 5 — Sup. Figure S5 [file 41418_2020_547_MOESM5_ESM.tif]
