# Supplementary material for: R-spondin2 signaling is required for oocyte-driven intercellular communication and follicular growth
Source: Cell Death Differ. 2020 Apr 27;27(10):2856–71. doi: 10.1038/s41418-020-0547-7 (PMC7493947; doi:10.1038/s41418-020-0547-7)
Supplement: Supplementary file 6 — Sup. Figure Table 1 [file 41418_2020_547_MOESM6_ESM.docx]

**Supplementary Table 1.** Primers used for qRT-PCR analyses

| Gene | Forward primer | Reverse primer |
| --- | --- | --- |
| *Alk4* | agagggtggggaccaaac | tgcttcatgttgattgtctcg |
| *Bmpr2* | gagccctcccttgacctg | gtatcgaccccgtccaatc |
| *Ccnb1* | CGCTGAAAATTCTTGACAACG | TCTTAGCCAGGTGCTGCATA |
| *Ccnd1* | GAGATTGTGCCATCCATGC | CTCCTCTTCGCACTTCTGCT |
| *Cdkn1a* | AACATCTCAGGGCCGAAA | TGCGCTTGGAGTGATAGAAA |
| *Cdkn1b* | GTTAGCGGAGCAGTGTCCA | TCTGTTCTGTTGGCCCTTTT |
| *Foxl2* | GGCGTCGTGAACTCCTACA | TGCAGATGATGTGCGTGAG |
| *Gdf9* | CCCAGCAGAAGTCACCTCTA | CAGGTTAAACAGCAGGTCCA |
| *KitL* | CGGCTCTCATTTCGCTTGTA | TGCCCTTGTAAGACTTGACTG |
| *cKit* | GGTACATGGCTGCATTCTGA | TTTCAGGCACAGACACAACA |
