# Supplementary material for: R-spondin2 signaling is required for oocyte-driven intercellular communication and follicular growth
Source: Cell Death Differ. 2020 Apr 27;27(10):2856–71. doi: 10.1038/s41418-020-0547-7 (PMC7493947; doi:10.1038/s41418-020-0547-7)
Supplement: Supplementary file 7 — Supplementary Figures Legends [file 41418_2020_547_MOESM7_ESM.docx]

**R-spondin2 signaling is required for oocyte-driven intercellular communication and follicular growth**

Marie-Cécile De Cian^1,2^, Elodie P. Gregoire^1^, Morgane Le Rolle^1^, Simon Lachambre^1^, Magali Mondin^3^ Sheila Bell^4^, Céline J. Guigon^5^, Anne-Amandine Chassot^1^ and Marie-Christine Chaboissier^1,&^.

**Supplementary information**

**Table1. List of PCR primers used for qRT-PCR analyses**

**Figure S1. Absence of RSPO2 impairs primary to secondary follicle stage transition**

(A) Immunolocalization of RSPO2 in a 12 month-old little girl ovary shows that RSPO2 is expressed in the cytoplasm of the oocyte in primordial (arrowhead), primary (arrow) and preantral (*) follicles. Scale bar, 50 µm. (B) In mice, histological sections (HE staining) analyses of wildtype (WT) ovaries at 21d reveal columnar granulosa cells with a basal localization of their nuclei in follicles containing one layer of granulosa cells. In two-layered follicles, nuclei of the external granulosa cells are localized close to the basal lamina whereas the granulosa nuclei of the internal layer are localized close to the oocyte. In contrast, granulosa cells do not exhibit the characteristic columnar shape of mature primary follicles in *Rspo2^Tg/Tg^* ovaries; they have a cuboidal shape with centered nuclei. Flattened granulosa cells are also observed in mutant primary follicles (*). The follicles with multi-layered granulosa cells demonstrate asymmetrical granulosa cell growth (arrows). Scale bar, 20µm. (C) Quantification of **Pr**, primordial; **P**, primary; **S**, secondary; **T**, tertiary; **A**, antral follicles in wildtype (black histograms) and *Rspo2^Tg/Tg^* (striped histograms) ovaries at 8, 12 and 21d. The numbers are percentages of the different follicle populations. At 8d, the percentages of primordial, primary and secondary follicles are similar in both WT and *Rspo2^Tg/Tg^* ovaries, but from 12d, secondary follicles are less abundant in *Rspo2^Tg/Tg^* ovaries (23.5% compared to 53.8% in WT). At 21d, mutant ovaries are still constituted of 62% primary follicles whereas WT ovaries mainly contain secondary and tertiary growing follicles. Data are presented as mean ± SEM. For WT, n=131(8d), 149 (12d), 133 (21d) and for *Rspo2^Tg/Tg^*, n=120 (8d), 132 (12d), 118 (21d) follicles from at least 3 different mice per genotype. Student's t test, unpaired two-sided (*p<0.05, **p<0.01, ***p< 0.001).

**Figure S2. Granulosa cells lose their identity in absence of RSPO2**

(A) Immunodetection analyses of FOXL2 and AMH (granulosa cells) and SF1/NR5A1 (theca cells) in WT and *Rspo2^Tg/Tg^* transplanted ovaries at 12d. Granulosa cells expressed FOXL2 and AMH but AMH expression is reduced in *Rspo2^Tg/Tg^* ovaries compared to wildtypes (WT). The presence of SF1/NR5A1 (theca)-positive cells was observed in both genotypes. (B) Immunodetection analyses of FOXL2 and AMH in control (*Ctnnb1^fl/fl^*) and mutant (*Wt1Cre^ERT2^;Ctnnb1^fl/fl^*) follicles at 12d*pp.* AMH expression is impaired in mutant ovaries compared to control. Scale bar, 50µm.

**Figure S3. Transplantation does not hamper follicular growth in wildtype ovaries.**

Quantitative analysis of the diameter size of oocytes (A) and follicles (B) in ovaries at 21d*pp* (White histograms; n=15 follicles of 1 ovary) and in transplanted ovaries at 21d (black histograms; n=55 follicles of 3 ovaries). **Pr**, primordial; **P**, primary; **S**, secondary; **T**, tertiary; **A**, antral follicles. Data are presented as mean ± SEM.

**Figure S4. Oocyte-granulosa cell communication is altered by genetic deletion of *Ctnnb1* in granulosa cells**

(A) Immunodetection and (B) corresponding quantitative analysis of nuclear versus cytoplasmic CTNNB1 in granulosa cells to assess activation of WNT/CTNNB1 signaling in control (*Ctnnb1^fl/fl^*) and mutant (*Wt1Cre^ERT2^;Ctnnb1^fl/fl^*) follicles at 21d*pp* (n=21 *Ctnnb1^fl/fl^* and n=26 *Wt1Cre^ERT2^;Ctnnb1^fl/fl^* follicles of 2 ovaries per genotype). In *Wt1Cre^ERT2^;Ctnnb1^fl/fl^* follicles, CTNNB1 expression is significantly decreased is the nuclei and the cytoplasm of granulosa cells. Data are presented as mean ± SEM. Student's *t* test, unpaired two-sided (***p<0.001). Scale bar, 20µm. (C) *In situ* hybridization analysis of *Axin2* expression and (D) corresponding quantification analysis highlighting a drastic down-regulation of *Axin2* in granulosa cells of *Wt1Cre^ERT2^;Ctnnb1^fl/fl^* follicles ~~but not in the oocyte~~ in comparison to control (*Ctnnb1^fl/fl^*) follicles at 21d*pp* (n=17 *Ctnnb1^fl/fl^* and n=20 *Wt1Cre^ERT2^;Ctnnb1^fl/fl^* follicles of 2 ovaries per genotype). Scale bar, 20µm. (E) QRT-PCR analysis of *Gdf9, Bmpr2 and Alk4* (GDF9 receptor), *Kit* receptor (*Kit*), *Kit* ligand *(KitL)* and *Foxl2* expression. Data are presented as individual data points. n= 3 ovaries per genotype. Mean values are indicated as black (*Ctnnb1^fl/fl^*) and grey (*Wt1Cre^ERT2^; Ctnnb1^fl/fl^*) bars.

**Figure S5. Image processing steps for *Axin2,* TJP1 and CDH2 signal intensity quantification.** (A) *Axin2* signal intensity was quantified on Z-stack images processed through sum slices projection and subsequent steps: a) gaussian blur on *Axin2* channel to determine oocyte Region Of Interest (ROI) and oocyte surface (*); b) manual drawing of follicle outline on DAPI channel; c) threshold adjustment of *Axin2* signal intensity; d) quantification of *Axin2* Raw Integrated Density within the oocyte ROI (*) and granulosa cell layer area (**) determined as the whole follicle area minus the oocyte surface.

(B) Quantification steps of TJP1 signal intensity after sum slices projection of Z-stack images : a) gaussian blur on Zona Pellucida (ZP1) channel to identify oocyte-granulosa cell interphase ROI (ZP1 ROI*); b) threshold adjustment of TJP1 signal intensity; c) manual drawing of follicle outline on CDH2 channel to determine granulosa cell surface as the whole follicle area minus the oocyte surface and the ZP1 ROI (GC ROI**); d) quantification of Apical TJP1 signal intensity, defined as Raw Integrated Density normalized on ZP1 ROI (µm^2^) and Lateral TJP1 signal intensity as Raw Integrated Density normalized on GC ROI (µm^2^). For CDH2 signal quantification, Z-stack images were processed through average intensity projection and signal intensity was quantified as Raw Integrated Density after threshold adjustment normalized on GC ROI (µm^2^).
